# Leishmaniasis in the Middle East: A Narrative Review of Epidemiological Characteristics and Transmission Determinants

**DOI:** 10.3390/pathogens15101093

**Published:** 2026-10-20

**Authors:** Basmah Alharbi, Mawahib Ahmed

**Affiliations:** 1 Department of Basic Health Sciences, College of Applied Medical Sciences, Qassim University, Buraidah 51452, Saudi Arabia; b.alwahbi@qu.edu.sa; 2 Department of Medical Laboratories, College of Applied Medical Sciences, Qassim University, Buraidah 51452, Saudi Arabia

**Keywords:** leishmaniasis, Middle East, cutaneous leishmaniasis, visceral leishmaniasis, *Leishmania major*, *Leishmania tropica*, sandfly, conflict, epidemiology, neglected tropical disease

## Abstract

Leishmaniasis is a neglected tropical disease (NTD) caused by protozoan parasites of the genus *Leishmania* and transmitted through phlebotomine sandfly bites. The Middle East carries a disproportionate share of the global burden, shaped by armed conflict, ecological change, population displacement, and fragmented healthcare infrastructure. This narrative review synthesizes evidence from twenty-four peer-reviewed studies published between 2001 and 2026 on the epidemiological patterns, transmission determinants, and public health implications of leishmaniasis in the Middle East. Cutaneous leishmaniasis (CL) caused by *Leishmania major* and *Leishmania tropica* constitutes the dominant clinical burden, with Syria, Iraq, Iran, Lebanon, and Saudi Arabia reporting the highest case counts in the region. Visceral leishmaniasis (VL) caused by *Leishmania infantum* and *Leishmania donovani* contributes a smaller but significant burden in Iran, Iraq, and Yemen. Conflict-driven displacement, climatic variables, urbanization, poverty, and immunosuppression were identified as factors consistently associated with elevated transmission across the included studies, though the relative contribution of each factor could not be quantitatively established given the observational and surveillance-based nature of the available evidence. Most included studies relied on passive surveillance data, limiting the strength of conclusions that can be drawn. Significant gaps remain around HIV-VL co-infection, standardized cross-border surveillance, and treatment access in conflict-affected settings. Strengthening regional surveillance networks, expanding mobile diagnostic units, and integrating migrant health policies into national programs are among the public health responses identified across the included literature as warranting prioritization.

## 1. Introduction

Leishmaniasis is a vector-borne parasitic disease caused by protozoan parasites of the genus *Leishmania*, transmitted to humans through the bites of infected female phlebotomine sandflies [1]. The disease manifests in three principal clinical forms: cutaneous leishmaniasis (CL), mucocutaneous leishmaniasis (MCL), and visceral leishmaniasis (VL), each defined by distinct parasite species, clinical presentation, and disease outcome [2]. CL is the most common form globally, presenting as localized skin ulcers or nodules that may heal spontaneously but often leave permanent scars. MCL affects the mucous membranes of the nose, mouth, and throat, producing destructive lesions, and is rare in the Middle East. VL, also known as kala-azar, is the most severe form, involving systemic dissemination to the liver, spleen, and bone marrow, and is fatal if untreated [2]. According to the CDC Yellow Book 2026, leishmaniasis is currently endemic in 99 countries, with an estimated 600,000 to 1,000,000 new CL cases and 50,000 to 90,000 new VL cases occurring annually [3]. The World Health Organization (WHO) classifies it as a neglected tropical disease (NTD), a designation reflecting both its disproportionate impact on impoverished populations and the persistent gap between its burden and available research funding [4]. Current first-line treatments, primarily pentavalent antimonials, carry significant toxicity and limited efficacy in many endemic settings, while access to newer agents such as liposomal amphotericin B remains restricted across low-resource countries [5].

*Leishmania* parasites cycle between two morphological forms: the promastigote, which develops in the sandfly midgut, and the amastigote, which replicates within host macrophages following inoculation into the skin [6]. More than 20 *Leishmania* species infect humans, while over 90 sandfly species have been identified as vectors, belonging primarily to the genus *Phlebotomus* in the Old World [1]. In the Middle East, CL is caused predominantly by *Leishmania major* and *Leishmania tropica*. *L. major* predominates in rural and arid zones, where rodents of the genera *Meriones* and *Rhombomys* serve as the primary zoonotic reservoir hosts, and *Phlebotomus papatasi* acts as the principal vector [7]. *L. tropica* is associated with urban and peri-urban transmission, sustained through anthroponotic cycles by *P. sergenti* in densely populated areas across Iran, Syria, Iraq, and neighboring countries [6]. VL in the region is caused primarily by *L. infantum*, with domestic dogs serving as the main reservoir and *P. perniciosus* and related species as vectors in Mediterranean-adjacent settings [7]. This ecological separation between CL-causing and VL-causing species has direct implications for control strategy: anthroponotic transmission can in principle be interrupted through case detection and treatment, while zoonotic cycles require broader ecological and veterinary interventions [8].

Host immune responses shape the clinical outcome of *Leishmania* infection and are relevant to understanding why disease severity varies across populations and settings. Protective immunity depends on a Th1 response involving interferon-gamma (IFN-gamma) and tumor necrosis factor-alpha (TNF-alpha), which activate macrophages to clear intracellular parasites [9]. Th2-associated cytokines, particularly interleukin-10 (IL-10), suppress this response and promote parasite persistence [10]. Immunosuppression from HIV co-infection, malnutrition, or prolonged corticosteroid use substantially worsens disease outcome and increases the risk of treatment failure, a dimension of particular relevance in the conflict-affected populations of the Middle East where malnutrition is prevalent and HIV testing coverage is limited [11,12].

Transmission patterns across the Middle East are also shaped by climatic and ecological variables. Rainfall, humidity, and temperature fluctuations influence sandfly population dynamics and seasonal transmission intensity [13]. In Saudi Arabia, CL incidence is significantly higher during winter months, consistent with the activity cycles of sandfly vectors and shifts in rodent reservoir density [14]. Urban expansion has introduced settled populations into areas that previously served as vector habitats, blurring the traditional boundary between rural zoonotic and urban anthroponotic transmission [8]. Cross-border ecological continuity and shared vector habitats among neighboring countries facilitate sustained movement of both vectors and reservoir hosts, making nationally siloed surveillance and control strategies insufficient [7].

The Middle East carries a substantial share of the global leishmaniasis burden. Afghanistan, Iran, and Syria together account for 70 to 75 percent of global CL incidence annually [15]. In Iran, over 20,000 CL and VL cases are reported each year across 17 provinces [8]. In Saudi Arabia, epidemiological mapping across all 13 administrative regions identified CL in both rural and urban settings, with non-Saudi residents accounting for 87 percent of confirmed cases, reflecting occupational and housing exposure in migrant worker populations [14]. Yemen carries a significant but underreported VL burden caused by *L. donovani*, where transmission intersects with active conflict and severe malnutrition, creating conditions for high case fatality [7]. In the Arabian Peninsula, VL transmission is primarily mediated by *Phlebotomus orientalis* and *Phlebotomus alexandri*, which differ ecologically from the *P. perniciosus* complex associated with *L. infantum* VL in Mediterranean settings [7,9]. The presence of these distinct vector species, adapted to the hot and arid conditions of the Arabian Peninsula, underlines the need for region-specific vector surveillance strategies that account for local sandfly ecology rather than applying Mediterranean-derived control models.

The Syrian conflict, which began in 2011, has had the most pronounced regional effect on leishmaniasis epidemiology. Population displacement into areas with established sandfly habitats, combined with the collapse of vector control programs and diagnostic infrastructure, generated CL outbreaks of unprecedented scale in Lebanon, Jordan, and Turkey [16,17]. CL cases in Syria reached over 50,000 annually at the height of the conflict, a figure that substantially underestimates the true burden given the near-total disruption of the national surveillance system [18]. Recent evidence confirms that elevated CL transmission in Syria has persisted beyond the peak conflict years, driven by continued displacement, inadequate sanitation in informal settlements, and the absence of sustained vector control measures [19]. These dynamics illustrate how political instability and forced migration act as force multipliers for leishmaniasis transmission in an already endemic region.

This narrative review consolidates evidence from studies published between 2001 and 2026 on the epidemiological patterns, transmission determinants, and public health implications of leishmaniasis in the Middle East. It identifies recurring knowledge gaps around HIV co-infection, standardized surveillance, and cross-border vector dynamics, and provides a regionally grounded basis for evidence-informed public health priorities.

## 2. Methodology

### 2.1. Study Design

This study adopts a narrative review approach to synthesize available evidence on the epidemiological patterns and transmission determinants of leishmaniasis in the Middle East. Narrative reviews are well suited to this objective because they allow interpretative synthesis across heterogeneous study designs, geographic settings, and epidemiological contexts. Rather than applying the fixed protocol-driven selection of a systematic review, this approach prioritizes breadth of coverage and thematic organization to identify key patterns, knowledge gaps, and public health implications relevant to the region.

### 2.2. Data Sources and Search Strategy

A literature search was conducted across PubMed, Scopus, Web of Science, Embase, and Google Scholar. Search terms combined Medical Subject Headings (MeSH) terminology and free-text keywords using Boolean operators. The primary search string applied was: *(Leishmaniasis OR “Cutaneous leishmaniasis” OR “Visceral leishmaniasis” OR “Leishmania”) AND (“Middle East” OR “Saudi Arabia” OR “Iran” OR “Iraq” OR “Syria” OR “Yemen” OR “Lebanon” OR “Jordan” OR “Turkey” OR “Afghanistan” OR “Kuwait” OR “Oman” OR “United Arab Emirates” OR “Palestine” OR “Israel”) AND (“epidemiology” OR “incidence” OR “prevalence” OR “risk factors” OR “vector control” OR “transmission” OR “burden”).* The geographic search terms were designed to capture primary burden countries in the Middle East region while remaining broad enough to include neighboring and ecologically comparable settings. Studies from countries not named in the search string but identified through reference list screening were also considered for inclusion where they were relevant to the review objectives.

Database-specific search approaches were applied where relevant. In PubMed, MeSH terms were used as the primary search framework, with free-text keyword searches applied in parallel to capture literature not indexed under standard MeSH. In Scopus and Web of Science, title, abstract, and keyword fields were searched using equivalent terminology. In Google Scholar and Embase, free-text searches were conducted using the same core terms. No language restrictions were applied during the search process, though all studies ultimately selected for inclusion were published in English. The search covered literature published from 2001 through 2026 to capture both foundational epidemiological data and the most recent evidence available at the time of writing. Grey literature, conference abstracts, and editorials were excluded.

### 2.3. Study Selection

Articles were selected based on relevance to the review objectives, geographic scope, and the quality of reported epidemiological data. Studies were included if they: (1) reported on the prevalence, incidence, risk factors, or public health response to leishmaniasis in Middle Eastern or directly comparable regional settings; (2) were published in peer-reviewed journals; and (3) provided sufficient methodological detail to assess the reliability of their findings. Studies focused exclusively on molecular mechanisms without epidemiological context were excluded, as were those unrelated to the defined regional scope. Studies conducted outside the Middle East were eligible for inclusion as comparative references only, and only where all three of the following conditions were met: the study addressed a *Leishmania* species, vector genus, or reservoir system also present in the Middle East; the epidemiological or ecological mechanism it documented had a direct analogue in a Middle Eastern setting for which comparable primary data were unavailable; and no Middle Eastern study of equivalent methodological quality addressed the same mechanism. Two studies met these criteria. Al-Salem et al. was included because conflict-associated VL caused by *L. donovani* in South Sudan reproduces the transmission conditions documented in Yemen and parts of Iraq, where surveillance collapse has prevented the generation of comparable primary data. Toumi et al. was included because its ecological setting in central Tunisia, defined by arid climate, zoonotic *L. major* transmission, and rodent reservoir dynamics, parallels adjacent Middle Eastern foci and provides the most robust quantitative rainfall-transmission data available for this cycle. No other non-regional studies met these criteria. Both are reported separately in Table 1, are discussed as comparators rather than as primary regional evidence, and are excluded from all regional burden figures. Reference lists of included articles were also reviewed to identify additional relevant sources not captured in the primary database search. The relatively limited number of studies selected reflects the genuine scarcity of rigorous primary epidemiological data from several countries in the region, particularly Yemen, Iraq, and Jordan, where conflict has disrupted both disease surveillance and research activity. Studies relying exclusively on hospital case series without population denominators were excluded to maintain methodological consistency. Grey literature reports and publications in languages other than English were also excluded; it is acknowledged that this approach may have missed relevant epidemiological data, particularly from conflict-affected countries where peer-reviewed publication activity is limited and local-language and grey literature sources may represent the primary available evidence. The included studies were selected to represent the breadth of clinical forms, geographic settings, and transmission determinants across the region rather than to achieve an exhaustive count of all available publications. With the addition of eight further studies identified during the revision process, the total number of included studies is twenty-four, comprising twenty-two studies reporting primary data from Middle Eastern settings and two external comparative studies presented separately in Table 1.

### 2.4. Approach to Synthesis

Findings were synthesized thematically rather than quantitatively. Each included study was read in full and assigned to one or more recurring determinants of transmission: conflict and population displacement, climatic and ecological conditions, socioeconomic vulnerability, immunosuppression, and surveillance capacity. Within each theme, reported associations were appraised against the study design that produced them, with particular attention to whether the underlying data were population-based, drawn from hospital case series, or derived from passive notification systems. Where studies reported divergent findings, the ecological and methodological reasons for divergence were examined rather than reconciled into a single summary statement. Consistent with the narrative design of this review, no formal risk of bias assessment, quality scoring, or quantitative pooling was undertaken, and no summary effect estimates are reported.

## 3. Results

A total of twenty-four studies were identified through database searching and reference list screening, of which twenty-two reported primary data from Middle Eastern settings and two were retained as external comparative references (Table 1), selected on the basis of their relevance to the epidemiological patterns, transmission determinants, and public health context of leishmaniasis in the Middle East and directly comparable regions. The included studies used varied research designs, encompassing retrospective observational studies, narrative reviews, epidemiological analyses, and ecological correlation studies. Populations examined ranged from conflict-affected refugee communities to national surveillance datasets. Recurring themes across the literature included the impact of armed conflict on disease transmission, climate and ecological drivers of vector activity, socioeconomic vulnerability, and the limitations of existing surveillance and control infrastructure. Table 1 provides a detailed summary of the characteristics of all included studies.

**Table 1:** Summary of Studies Included in the Narrative Review on Leishmaniasis in the Middle East and Comparable Settings.

Study Location	Type of *Leishmania*	Study Design	Study Population	Key Findings
**Lebanon** **[16]**	Cutaneous (*L. tropica*)	Retrospective observational study	Displaced Syrian refugees in Lebanon	Conflict-induced displacement contributed to a sharp rise in CL cases; 96.6% of cases were among Syrian nationals
**Saudi Arabia, Iraq, Syria** **[8]**	Cutaneous (*L. major*, *L. tropica*)	Epidemiological analysis	Global and regional disease burden data	Revised CL burden estimates; underreporting identified as a major obstacle to accurate surveillance
**Saudi Arabia, Jordan, Oman** **[11]**	Cutaneous and Visceral	Narrative review	Regional incidence data across multiple countries	Documented underreporting and the role of regional conflict in driving transmission
**Iran** **[6]**	Cutaneous (*L. major*, *L. tropica*), Visceral (*L. infantum*)	Systematic review	Multiple Iranian provinces	Expansion of CL foci into urban zones; over 20,000 annual cases across 17 provinces
**Iraq, Saudi Arabia, Yemen** **[9]**	Cutaneous (*L. tropica*, *L. major*)	Narrative synthesis	Recent regional epidemiological studies	Emerging CL foci in urbanized zones; limited healthcare access and diagnostic capacity
**Middle East and East Africa** **[20]**	Cutaneous (*L. tropica*, *L. major*), Visceral (*L. infantum*, *L. donovani*)	Narrative review	Human and animal populations	VL transmission in Yemen and the Arabian Peninsula mediated primarily by *P. orientalis* and *P. alexandri*, ecologically distinct from Mediterranean *L. infantum* vectors.
**Syria** **[18]**	Cutaneous (*L. tropica*, *L. major*)	Narrative review	National surveillance data, 2011–2018	CL cases reached over 50,000 annually at conflict peak; health system collapse impaired reporting
**Jordan** **[21]**	Cutaneous (*L. tropica*)	Retrospective study	Syrian refugees in Jordan	Significant rise in CL cases post-2011; *L. tropica* was the predominant species identified
**Lebanon** **[17]**	Cutaneous (*L. tropica*)	Retrospective observational study	Syrian refugees across multiple camps in Lebanon from September 2012	CL outbreak documented among Syrian refugees from September 2012; 948 patients enrolled for diagnostic confirmation; *L. tropica* identified in 85% and *L. major* in 15% of cases; initial treatment cure rate 82% after meglumine antimoniate
**Regional (Middle East and Mediterranean)** **[22]**	Cutaneous (*L. major*, *L. tropica*)	Narrative review	General population in endemic countries	Comprehensive overview of CL epidemiology, diagnosis, and treatment gaps in resource-limited settings
**Syria** **[19]**	Cutaneous (*L. tropica*, *L. major*)	Epidemiological review	Conflict-affected Syrian population, 2011–2024	Sustained high CL transmission confirmed beyond peak conflict years; displacement and absent vector control identified as primary drivers
**Iran** **[23]**	Cutaneous (*L. major*)	Retrospective registry-based study	Confirmed CL patients referred to CL Research Centers in Mashhad, northeastern Iran, 2015–2024	1537 confirmed CL cases identified; children under 10 accounted for 34.9% of cases; majority resided in peri-urban areas; male patients slightly more frequent; low educational level in 71.6% of cases
**Iraq** **[24]**	Cutaneous (*L. major*, *L. tropica*)	Retrospective epidemiological analysis	Civilian population across Iraqi provinces using Ministry of Health surveillance data, 2011–2013	CL burden documented across all provinces; higher case counts in lowland provinces with moderate annual rainfall and high rural populations; males and children aged 5–14 disproportionately affected
**Yemen** **[25]**	Cutaneous, Mucocutaneous, Visceral (*L. tropica*, *L. donovani*, *L. infantum*)	Cross-sectional observational study	152 patients with confirmed leishmaniasis at Regional Leishmaniasis Control Center, central Yemen, April–August 2013	Children and adult females at highest risk accounting for 90.1% of cases; MCL was most prevalent form (49.3%), followed by CL (47.4%) and VL (3.3%); Al Bayda most endemic governorate (59.9%); 94.1% of patients were rural residents
**Saudi Arabia** **[26]**	Cutaneous (*L. major*, *L. tropica*)	Retrospective observational study using Ministry of Health data	National CL case records, January 2012 to December 2023	CL incidence peaks during winter months; 12-year trend shows no sustained reduction despite national control efforts; seasonal pattern consistent with *P. papatasi* activity cycles
**Saudi Arabia** **[27]**	Cutaneous (*L. tropica*, *L. major*)	Retrospective observational study	CL patients in Al-Qassim region, 2016–2023	Seasonal clustering confirmed; male patients and adults aged 20–40 at highest risk; peri-urban settings predominate; *L. tropica* most frequent species identified
**Iran** **[28]**	Cutaneous (*L. major*, *L. tropica*)	Situation analysis with SARIMA time series modeling	Provincial CL surveillance data, 2013–2020	Cyclical CL incidence pattern with peaks in 2014–2015 and predicted recurrence in 2022–2023; urban expansion and climate variability identified as key drivers; findings support national elimination planning
**Iran** **[29]**	Visceral (*L. infantum*)	Cross-sectional seroprevalence study with narrative review	National VL seroprevalence data from 10 provinces, 2013–2022	Human and canine VL seroprevalence documented across northwestern and central provinces; gradual decline in human cases but persistent canine reservoir sustains ongoing transmission risk
**Iraq** **[30]**	Cutaneous (*L. major*, *L. tropica*)	Retrospective six-year molecular epidemiological study	2325 suspected CL cases in Al-Muthanna province, 2015–2020	*L. tropica* and *L. major* confirmed by ITS1 gene sequencing; males and young adults most affected; highest burden in spring and summer; both species co-circulate in southern Iraq
**Turkey** **[31]**	Cutaneous (*L. tropica*, *L. major*)	Epidemiological review using WHO and OIE case data	National CL records across Turkey, 2005–2020	CL incidence peaked at 7.02 per 100,000 in 2013 driven by mass refugee influx from Syria; *L. tropica* dominant in imported cases; epidemic curve directly linked to Syrian conflict and cross-border population movement
**Middle East Multi-country** **[32]**	Cutaneous (*L. major*, *L. tropica*, *L. infantum*)	Systematic review and meta-analysis	Studies from 16 Middle Eastern countries through June 2021	Highest CL prevalence in Syria (39%); data available for Bahrain, Cyprus, Egypt, Iran, Iraq, Jordan, Kuwait, Lebanon, Oman, Palestine, Qatar, Saudi Arabia, Syria, Turkey, UAE, and Yemen; significant heterogeneity across settings
**Qatar** **[33]**	Cutaneous and Visceral	Retrospective cross-sectional study	68 confirmed leishmaniasis cases at Hamad Medical Corporation, 2016–2022	Majority of cases among expatriate workers; CL predominant; Yemen reported 6416 CL cases from 2019 to 2022; Syria reported 89,357 cases in 2019; Qatar data illustrate the mobile burden across the Gulf region

Epidemiological data from studies reporting specific case numbers are summarized in Table 2. The figures presented reflect considerable variation across countries, attributable to differences in surveillance capacity, conflict intensity, and healthcare infrastructure rather than true differences in disease burden alone. The data consistently point to conflict-driven surges in both CL and VL across the region, with Syria, Lebanon, and Iran carrying the highest documented burdens. Table 2 separates population-based incidence data from hospital-based case series and passive notification registry data, because these source types carry materially different implications for interpreting reported case counts. Population-based figures permit incidence estimation against a defined denominator, whereas case series and passive notification data reflect both true disease occurrence and the diagnostic and reporting capacity available at the point of ascertainment.

Three broad categories of risk factors emerged consistently across the included studies: armed conflict and population displacement, climatic and ecological variables, and socioeconomic conditions including poverty and inadequate housing. These factors are rarely independent in most settings, they interact and reinforce one another, creating compounded transmission risk. Table 3 presents the specific risk factors and environmental determinants identified across included studies.

The included studies described or recommended a range of public health interventions, varying considerably by country, conflict status, and available resources. Emergency responses were most commonly documented in conflict-affected settings, while integrated ecological and surveillance-based approaches were more prominent in stable countries with established health infrastructure. Table 4 summarizes the interventions and their reported outcomes across included studies.

Two studies conducted outside the Middle East were retained as external comparative references on the basis of specific epidemiological or ecological parallels to the region. These are presented separately in Table 5 and are excluded from the regional burden figures reported in Table 1, Table 2, Table 3 and Table 4.

These studies are cited as comparators only. Their case counts and incidence figures are not incorporated into any regional burden estimate presented in this review.

## 4. Discussion

This narrative review examined the epidemiological patterns and transmission determinants of leishmaniasis in the Middle East, drawing on twenty-four studies published between 2001 and 2026. CL caused by *L. major* and *L. tropica* remains the dominant form of leishmaniasis across the Middle East, with Syria, Iraq, Iran, Lebanon, and Saudi Arabia reporting the highest case burdens in the region [8,18,19]. The evidence synthesized in this review identifies a consistent set of factors associated with elevated leishmaniasis transmission across countries and settings: armed conflict and population displacement, climatic and ecological change, socioeconomic vulnerability, and weak surveillance infrastructure. The available studies are predominantly observational and rely on passive surveillance data, which limits the ability to establish causal relationships or quantify the independent contribution of any single factor to observed changes in disease burden. These factors do not operate independently. In most affected settings they interact and reinforce one another, making single-intervention control strategies largely inadequate. Of the drivers identified across included studies, three were reported consistently across multiple countries and settings: conflict-driven population displacement, socioeconomic vulnerability including poverty and inadequate housing, and climatic variables, particularly seasonal rainfall and temperature fluctuations influencing sandfly activity. These three factors appeared in studies from Syria, Lebanon, Jordan, Iran, Saudi Arabia, and South Sudan regardless of the specific *Leishmania* species or clinical form under study [8,13,14,18,19]. Among these, socioeconomic and conflict-related factors are supported by the most consistent observational evidence. The finding that non-Saudi residents accounted for 87 percent of confirmed CL cases in Saudi Arabia [14] and that CL rates among Syrian refugees in Lebanon reached 96.6 percent of all reported cases [16] provides comparatively direct epidemiological support for the role of displacement and poverty in shaping disease burden. Climatic associations, while biologically plausible and supported by seasonal data from Saudi Arabia [14] and ecological data from Tunisia [13], rest on correlational evidence from observational studies and should therefore be interpreted as supporting rather than establishing causality.

Armed conflict is the factor most consistently associated with documented surges in CL transmission across the region, appearing as a contextual determinant in studies from Syria, Lebanon, Jordan, Iraq, and South Sudan, though its independent causal contribution cannot be separated from co-occurring factors such as population movement, healthcare collapse, and surveillance disruption. The Syrian conflict, which began in 2011, generated CL outbreaks of a scale not previously recorded in Lebanon, Jordan, and Turkey, as displaced populations moved into sandfly habitats while vector control programs collapsed [17,18]. CL cases in Syria reached over 50,000 annually at the conflict peak, a figure that substantially underestimates true incidence given the near-total disruption of the national health system [18]. Elevated transmission has persisted well beyond the acute conflict phase, driven by continued displacement, inadequate sanitation in informal settlements, and the absence of vector control programs [19]. A comparable association between conflict and VL burden was documented in South Sudan, where *L. donovani* outbreaks coincided with displacement and health system collapse, offering a contextually relevant parallel to conditions in Yemen and parts of Iraq, though differences in parasite species, vector ecology, and population immunity limit direct comparability [34]. Disentangling genuine transmission increases from improved case detection or population movement is a core interpretive challenge for this review. In Syria, the documented surge to over 50,000 annual CL cases during the conflict peak [18] reflects a combination of factors that cannot be fully separated with the available data: displacement of non-immune populations into sandfly habitats, collapse of vector control allowing vector populations to expand, and concentration of cases in settings with functional surveillance such as refugee health facilities. In Lebanon and Jordan, some of the documented increase in cases following 2011 is attributable to the arrival of Syrian refugees who had already acquired infection in Syria rather than to new local transmission events [16,17,21]. Case detection capacity also varied substantially. Areas with active humanitarian health programs recorded more cases precisely because surveillance was stronger, not necessarily because transmission was higher. These interpretive limits mean that raw case counts across conflict-affected settings should be read as indicative of burden concentration rather than as direct measures of transmission intensity.

Climatic and ecological variables form a second major category of transmission determinants. Rainfall, humidity, and temperature fluctuations influence sandfly population dynamics and the density of rodent reservoir hosts, directly affecting the intensity and seasonality of CL transmission [13]. In Saudi Arabia, CL incidence follows a clear seasonal pattern with higher case counts during winter months, consistent with the activity cycles of *P. papatasi* and fluctuations in rodent reservoir density [14]. Urbanization has added complexity to transmission geography, with *L. tropica* now documented in densely populated urban centers across Iran, where sandfly vectors have adapted to peri-domestic environments created by urban expansion and land-use change [6]. In central Tunisia, a positive correlation between rainfall, humidity, and zoonotic CL incidence was documented through increased rodent reservoir proliferation and sandfly breeding activity [13]. Tunisia falls outside the geographic scope of this review, but its ecological conditions arid climate, *L. major* transmission, and rodent reservoirs parallel those in adjacent Middle Eastern zones and offer useful comparative insights, provided direct extrapolation is made with appropriate caution. Taken together, these patterns support the development of climate-informed surveillance systems capable of anticipating seasonal transmission peaks rather than responding reactively to reported cases. The consistency of climatic associations across ecological settings is partial rather than uniform. Rainfall-driven surges in CL incidence are documented in the arid zones of central Tunisia through zoonotic *L. major* cycles [13], and seasonal winter peaks in CL are reported in Saudi Arabia through *P. papatasi* activity cycles [14]. However, in urban settings across Iran, temperature rise and habitat modification associated with urbanization appear more relevant than rainfall alone, reflecting the different transmission dynamics of *L. tropica* in anthroponotic urban cycles [6]. This suggests that the direction and magnitude of climatic influence on CL transmission depends substantially on the local species composition, vector ecology, and land-use context, and that a single climate-transmission model cannot be applied uniformly across the Middle East. The relative contribution of *L. major* and *L. tropica* does differ systematically between rural and urban settings. *L. major* predominates in rural and arid zones, sustained through zoonotic cycles involving *P. papatasi* and rodent reservoirs of the genera *Meriones* and *Rhombomys* [7]. Its transmission is therefore driven primarily by ecological and climatic conditions that support reservoir and vector populations. *L. tropica*, by contrast, is predominantly urban and sustains transmission through anthroponotic cycles involving *P. sergenti* in densely populated areas [6]. Studies from Iran confirm that urbanization has expanded *L. tropica* foci into cities where *L. major* was previously absent or rare [6]. This rural-urban species divide has direct implications for control: *L. major* requires ecological and veterinary interventions targeting reservoir populations, while *L. tropica* can in principle be controlled through early case detection and treatment given its human-to-human transmission cycle [8].

Socioeconomic conditions interact with ecological and conflict-driven factors to sustain transmission in ways that are consistent across settings. Populations living in poverty, informal settlements, or active conflict zones face restricted access to diagnostics and treatment, which creates conditions where infection remains undetected and untreated for extended periods [8,11]. Migrant and refugee populations carry a disproportionate share of the burden in several countries. In Saudi Arabia, non-Saudi residents accounted for 87 percent of confirmed CL cases between 2020 and 2022, reflecting occupational exposure, substandard housing conditions, and limited access to preventive care among migrant worker communities [14]. The cross-sectional and retrospective designs used in most included studies limit causal inference. The observed associations between poverty, displacement, and leishmaniasis incidence reflect risk concentration in vulnerable populations rather than established causal pathways and should be interpreted with this methodological limitation in mind.

HIV co-infection and immunosuppression represent an underappreciated dimension of leishmaniasis burden in the Middle East that the current evidence base addresses only superficially. In HIV-positive individuals, VL follows a more aggressive clinical course with higher rates of treatment failure and relapse, driven by the loss of effective Th1-mediated immunity [12]. Treatment outcomes are further worsened by high relapse rates following standard amphotericin B regimens and the absence of region-specific clinical guidelines for managing co-infected patients in resource-limited settings. In the Middle East, where HIV testing coverage is limited and healthcare access is disrupted by ongoing conflict, the true burden of HIV-VL co-infection is likely substantially underestimated [11]. Immunosuppression from malnutrition, which is prevalent in conflict-affected populations across Yemen, Syria, and Iraq, similarly alters disease presentation and reduces treatment response [12]. Dedicated prospective studies addressing HIV-leishmaniasis co-infection specifically in the Middle Eastern context are absent from the current literature and represent a clear and addressable research priority.

Treatment access and diagnostic capacity remain significant barriers to effective disease control across the region. Pentavalent antimonials, the traditional first-line therapy for CL and VL, carry substantial toxicity and are associated with growing treatment resistance in several endemic areas [5]. Liposomal amphotericin B offers improved safety but remains largely inaccessible in the low-resource settings where the disease burden is highest. In conflict-affected countries, the collapse of laboratory infrastructure has made basic parasitological diagnosis unavailable in many areas, leaving clinicians to rely on clinical judgment alone [9,20]. Point-of-care diagnostics, including rapid diagnostic tests based on rK39 antigen detection for VL, have shown practical value in resource-limited settings but have not been systematically deployed across the Middle East [20]. Mobile clinical units used during the South Sudan VL outbreak demonstrated that decentralized treatment delivery can meaningfully improve care access in active conflict zones, providing a model relevant to Middle Eastern settings with similar constraints [34].

Surveillance gaps cut across all the drivers discussed above and constrain the ability to draw firm conclusions from the available evidence. The majority of included studies relied on passive case reporting systems, which capture only a fraction of true incidence in settings with limited laboratory capacity and restricted healthcare access [8,11]. Case definitions varied across countries and study periods, making direct comparisons of incidence trends unreliable. The absence of standardized cross-border surveillance data is a structural gap that no individual country study can resolve in isolation. Regional cooperation mechanisms, including shared case definitions, cross-border vector surveillance, and coordinated outbreak response, are consistently identified as priorities across the included literature but remain poorly implemented in practice [9,22]. Building a regional leishmaniasis surveillance network through existing WHO Eastern Mediterranean Regional Office frameworks would represent a practical and meaningful step toward addressing this gap.

A further methodological limitation concerns the comparison of epidemiological findings across settings with distinct parasite, vector, and reservoir complexes. *L. major* transmission in rural arid zones operates through zoonotic cycles involving *P. papatasi* and rodent reservoirs of the genera *Meriones* and *Rhombomys*, whereas *L. tropica* sustains transmission through anthroponotic urban cycles involving *P. sergenti* with no established animal reservoir. VL transmission is similarly heterogeneous: *L. infantum* in Mediterranean-adjacent settings is maintained by the *P. perniciosus* complex with domestic dogs as the principal reservoir, while VL in the Arabian Peninsula and Yemen is mediated primarily by *P. orientalis* and *P. alexandri* under hot and arid conditions, with *L. donovani* transmission in these foci being largely anthroponotic. Incidence estimates, seasonality patterns, and risk factor associations generated within one of these complexes are therefore not directly transferable to another. This constraint applies with particular force to the two external comparators retained in this review. The rainfall-incidence relationship documented for zoonotic *L. major* in central Tunisia depends on a rodent reservoir cycle absent from anthroponotic *L. tropica* foci, and the conflict-driven VL dynamics described in South Sudan involve *P. orientalis* mediated *L. donovani* transmission that differs ecologically from *L. infantum* VL in Iran and Iraq. Comparative inferences drawn from these settings should therefore be read as indicating plausible mechanisms rather than as transferable quantitative estimates, and the same caution applies to cross-country comparison within the region wherever the dominant species or vector differs.

Several methodological limitations of this review warrant explicit acknowledgment. The search was restricted to studies published in English, which likely resulted in the exclusion of relevant epidemiological data published in Arabic, Persian, and Turkish the primary scientific languages of several high-burden countries in the region. Studies from Iran, Iraq, Syria, and Yemen published in national or regional journals in local languages were not captured and may contain primary surveillance data not available in English-language literature. Grey literature was also excluded from this review, including World Health Organization country reports, bulletins from regional health ministries, and data compiled by non-governmental organizations operating in conflict zones. In settings such as Syria, Yemen, and Iraq, where academic research activity has been severely curtailed by ongoing conflict, grey literature often represents the only available source of disease burden data. Its exclusion means that the case counts presented in this review, while drawn from the best available peer-reviewed evidence, likely underestimate the true regional burden of leishmaniasis. For these reasons, this review is best understood as a structured synthesis of published transmission determinants and epidemiological patterns rather than as a definitive or complete accounting of regional disease incidence. Future narrative or systematic reviews of leishmaniasis in the Middle East should prioritize multilingual searches and incorporate grey literature sources to obtain a more complete picture of the burden.

Reducing the burden of leishmaniasis in the Middle East requires approaches that address clinical management alongside the structural and ecological conditions that sustain transmission. Expanding mobile diagnostic and treatment units in conflict-affected settings has demonstrated practical value and should be scaled in active outbreak zones [34]. Community-level vector control, including insecticide spraying targeting sandfly breeding sites and environmental modification to reduce rodent habitat, remains a core intervention, though its effectiveness is limited by growing insecticide resistance in *P. papatasi* populations documented across the region [8,22]. Migrant and refugee health policies in receiving countries need to incorporate leishmaniasis screening and treatment access, given the documented disproportionate burden in these populations [14,16]. Before translating findings into regional policy, it is important to distinguish between those that appear country-specific and those with broader regional applicability. The association between seasonal winter rainfall and CL incidence documented in Saudi Arabia [14] and the role of *L. tropica* urbanization in Iran [6] reflect country-specific ecological and demographic contexts that may not generalize directly to other settings. By contrast, the link between conflict-driven population displacement and surges in CL transmission has been documented consistently across Syria, Lebanon, Jordan, and South Sudan [16,17,18,19,34], suggesting this association is regionally generalizable and should inform cross-border public health planning. Similarly, the finding that migrant and refugee populations carry a disproportionate share of CL burden appears consistent across Lebanon [16,17], and Saudi Arabia [14], suggesting that targeted screening and treatment policies for these groups represent a generalizable regional priority. Future research should prioritize longitudinal vector surveillance data, standardized case definitions enabling cross-country comparison, and dedicated studies on HIV co-infection outcomes, treatment resistance patterns, and health system requirements for sustained control in post-conflict settings [9,19].

## 5. Conclusions

This narrative review draws on evidence from twenty-four studies to summarize the epidemiological patterns and transmission determinants of leishmaniasis in the Middle East. CL caused by *L. major* and *L. tropica* represents the dominant clinical burden across the region, with the highest case counts consistently reported from conflict-affected countries where population displacement, deteriorating housing conditions, and interrupted vector control programs are associated with elevated transmission. VL caused by *L. infantum* and *L. donovani* presents a smaller but significant burden, particularly in Iran, Iraq, and Yemen, where it intersects with malnutrition and active conflict to produce high case fatality rates. Climatic variables, including seasonal rainfall and temperature fluctuations, drive the density and activity of sandfly vectors and their rodent reservoir hosts, with clear implications for the seasonality and geographic distribution of disease. Socioeconomic vulnerability, including poverty, inadequate housing, and restricted access to diagnostics and treatment, is consistently associated with elevated transmission in both conflict-affected and stable settings, with migrant and refugee populations carrying a disproportionate share of the documented burden.

The evidence base is limited by the reliance of most included studies on passive surveillance data, heterogeneous study designs, and inconsistent case definitions across countries. These methodological constraints limit the strength of conclusions that can be drawn about true incidence trends and causal pathways. HIV co-infection, immunosuppression from malnutrition, and treatment resistance represent underaddressed dimensions of the regional disease burden that dedicated prospective studies are needed to characterize. Standardized surveillance systems, cross-border cooperation, and investment in point-of-care diagnostics are the clearest structural priorities emerging from this synthesis. Migrant and refugee health policies in receiving countries need to incorporate systematic leishmaniasis screening and treatment access. Future research should focus on longitudinal vector surveillance, the health system requirements for sustained control in post-conflict settings, and the development of region-specific clinical guidelines for managing immunocompromised patients with leishmaniasis.

## Figures and Tables

**Table 2:** Epidemiological Case Data on Leishmaniasis in the Middle East, Stratified by Data Source Type.

Country	Data Source Type	Clinical Form	Reported Case Numbers	Notable Findings	Incidence Trend
**Panel A. Population-based incidence data**					
**Saudi Arabia** **[14]**	National surveillance across all 13 administrative regions with population denominator	CL	2280 cases between 2020 and 2022	Non-Saudi residents accounted for 87% of cases; mapping covered both urban and rural settings	Consistent burden across urban and rural settings
**Iran** **[6]**	Aggregated provincial surveillance data across 17 provinces	CL (*L. tropica*, *L. major*), VL (*L. infantum*)	Over 20,000 cases annually	Urban and rural foci expanding across 17 provinces	Progressive increase linked to urbanization and ecological change
**Panel B. Hospital-based case series and passive notification registry data**					
**Syria** **[18]**	Passive national notification registry, 2011 to 2018	CL (*L. tropica*, *L. major*)	Over 50,000 cases annually at conflict peak	Surveillance collapse prevented accurate case ascertainment; reported figures represent a lower bound	Marked increase from 2011, sustained into the post-conflict period
**Lebanon** **[16]**	Passive notification data, Ministry of Public Health	CL (*L. tropica*)	1033 cases in 2013	96.6% of cases among Syrian refugees; highest recorded outbreak in Lebanon	Sharp rise after the onset of the Syrian conflict in 2011
**Lebanon** **[17]**	Clinic and laboratory-based case series across refugee camps, no population denominator	CL (*L. tropica*, *L. major*)	948 patients enrolled for diagnostic confirmation from September 2012; *L. tropica* in 85% and *L. major* in 15%	Initial treatment cure rate of 82% after three months of meglumine antimoniate	Ongoing epidemic documented from late 2012

Case numbers in Panel B are not directly comparable with those in Panel A, since they lack population denominators and are conditioned on local diagnostic and reporting capacity.

**Table 3:** Risk Factors and Environmental Determinants of Leishmaniasis Transmission in the Middle East.

Country	Clinical Form	Key Risk Factors Identified	Environmental or Climatic Influence
**Iran** **[6]**	CL (*L. tropica*, *L. major*), VL (*L. infantum*)	Urbanization, ecological change, land-use modification	Temperature rise and habitat expansion extended vector range into urban areas
**Saudi Arabia, Syria, Iraq** **[8]**	CL (*L. major*, *L. tropica*)	Conflict, poverty, population displacement, underreporting	Migration into high-risk endemic zones increased human exposure
**Saudi Arabia** **[14]**	CL	Migrant worker exposure, occupational and housing risk	Seasonal sandfly activity; higher winter incidence
**Syria** **[18]**	CL (*L. tropica*, *L. major*)	Armed conflict, surveillance collapse, displacement	Disruption of vector control programs during conflict
**Syria** **[19]**	CL (*L. tropica*, *L. major*)	Prolonged displacement, informal settlements, absent vector control	Continued ecological exposure in post-conflict informal zones

**Table 4:** Public Health Interventions and Control Measures for Leishmaniasis in the Middle East.

Country	Intervention Type	Key Findings	Outcomes and Gaps
**Lebanon** **[16]**	Passive surveillance, emergency vector control	CL outbreak among Syrian refugees triggered a local health response including insecticide spraying and monitoring	Partial containment achieved; response limited by diagnostic shortages and resource constraints
**Iran** **[6]**	Integrated ecological and urban management	Urbanization-driven CL expansion required integration of land-use planning with vector surveillance	Improved mapping of urban foci; full implementation of integrated control remains limited
**Middle East and East Africa** **[20]**	Integrated Vector Management, community engagement	IVM approaches identified as needed in rural and agricultural zones with absent conventional vector control	Community-driven strategies proposed; systematic regional implementation lacking
**Saudi Arabia, Syria, Iraq** **[8]**	Surveillance strengthening, policy reform	Disease burden substantially underreported; data gaps limit planning and resource allocation	Regional cooperation and standardized reporting systems identified as priorities
**Iraq, Saudi Arabia, Yemen** **[9]**	Diagnostic capacity building, healthcare access	Limited diagnostic infrastructure identified as a major barrier to case detection and treatment	Investment in point-of-care diagnostics and trained personnel identified as key needs

**Table 5:** External Comparative Studies Referenced for Epidemiological Context Outside the Middle East.

Study Location	Type of *Leishmania*	Study Design	Key Findings	Rationale for Comparative Inclusion
**South Sudan** **[34]**	Visceral (*L. donovani*)	Narrative review	VL outbreaks driven by displacement and collapse of health systems; over 7000 cases reported in 2014	Conflict-associated VL transmission closely mirrors conditions documented in Yemen and parts of Iraq, where equivalent primary surveillance data are unavailable because of conflict-related disruption of reporting systems
**Central Tunisia** **[13]**	Cutaneous, zoonotic (*L. major*)	Retrospective observational study	Rainfall and humidity strongly correlated with zoonotic CL incidence through increased rodent reservoir density and sandfly breeding activity	Arid climate, *L. major* transmission, and rodent reservoir ecology parallel adjacent Middle Eastern zones; provides the most methodologically robust available rainfall-transmission data for zoonotic *L. major*

## Data Availability

No new data were created or analyzed in this study. Data sharing is not applicable to this article.
